# Outcomes of Thin Groin Flaps for Hand and Finger Reconstruction: A Mixed Retrospective-Prospective Study

**DOI:** 10.7759/cureus.105911

**Published:** 2026-03-26

**Authors:** Yahia A Alsiaghi, Basheer M Othman, Mohaned Y Al-ajaly, Faisal A Ali, Gehad A Almashrmah, Ayman Ghanem, Mohammed A Saghir

**Affiliations:** 1 Department of Plastic Surgery, Typical Police Hospital, Sana'a, YEM; 2 Department of Plastic &amp; Reconstructive Surgery, Al-Gamhori Teaching Hospital, 21 September University, Sana’a, YEM; 3 Department of Plastic Surgery, Al-Gamhori Teaching Hospital, 21 September University, Sana’a, YEM; 4 Department of Orthopedic Surgery, Al-Thawra Modern General Hospital, Sana'a, YEM; 5 Faculty of Medicine and Health Sciences, University of Saba Region, Marib, YEM; 6 Department of Public Health and Epidemiology, University of Bahri, Khartoum, SDN; 7 Department of Orthopedic Surgery, Egyptian Board, Cairo, EGY

**Keywords:** flap, groin, hand, reconstruction, yemen

## Abstract

Introduction

Reconstruction of soft tissue defects in the hand and fingers remains challenging, particularly in complex injuries. The thin groin flap, a pedicled flap technique, offers a reliable option for coverage with acceptable functional and aesthetic outcomes.

Objective

The objective of the study is to evaluate the clinical and functional outcomes of thin groin flap reconstruction for hand defects.

Methods

This was a mixed retrospective-prospective study conducted in government and private hospitals in Sana'a, Yemen (Typical Police Hospital and Elite Hospital), which have reconstructive surgery units from January 2021 to December 2024. Outcomes assessed included flap healing time, operation duration, Disabilities of the Arm, Shoulder, and Hand (DASH) scores at one month, complication rates, and patient satisfaction. A subgroup analysis evaluated the impact of using magnification loupes during surgery.

Results

This study involved 25 patients; the median age was 27.0 years (interquartile range (IQR) 22.0-40.0) with a slight male predominance (14, 56.0%). Primary causes of injury were non-gunshot trauma (10, 40.0%), burns (10, 40.0%), and high-energy gunshot injuries (4, 16.0%). The median defect size was 7.0 cm² (IQR 6.0-7.0), and the median time from injury to surgery was 7.0 days (IQR 5.0-16.0). Surgical magnification loupes were used in 22 (88.0%) of procedures. A complete flap survival rate of 24 (96.0%) was achieved, with an overall success rate of 20 (80.0%). Functional outcomes, assessed by the DASH score, were generally favorable (median 10.0, IQR 5.0-25.0). Surgical magnification loupes significantly improved outcomes, reducing complication rates (9.1% (n = 2) vs. 100.0% (n = 3) in the no-loupe group (Fisher's exact test, p = 0.028)), improving functional scores (median DASH 10.0 vs. 50.0 (Mann-Whitney U = 0.0, p = 0.018)), and shortening hospital stays (median 4.5 days vs. 15.0 days; p = 0.004). Predictors of poorer functional outcomes included gunshot wounds, associated injuries, longer time from injury to surgery, and larger defect size.

Conclusion

The study concludes that thin groin flaps are a reliable option for hand reconstruction, with surgical magnification significantly enhancing outcomes and patient recovery.

## Introduction

Hand injuries are incredibly common, accounting for 6.6% to 28.6% of all musculoskeletal issues. They primarily affect working-class men under 40, often leading to significant economic impacts [1]. These injuries, which often involve complex soft tissue defects, can result from a variety of causes, including trauma, burns, tumor excision, congenital deformities, or accidents, particularly in conflict zones [2]. In such cases, timely and effective reconstruction is crucial to restore both the form and function of the hand, enabling patients to regain their independence and quality of life [1,3].

The reconstruction of hand injuries poses significant challenges due to the need for both soft tissue coverage and the preservation or restoration of underlying structures such as tendons, nerves, and blood vessels. Among the many reconstructive techniques available, the thin groin flap has gained increasing popularity due to its advantageous characteristics. As a modified version of the traditional groin flap, the thin groin flap offers a thinner, more pliable tissue suitable for complex hand reconstructions [4,5]. This technique provides several benefits, including minimal donor-site morbidity, adequate vascular supply, and favorable functional and aesthetic outcomes, making it an ideal option for patients who require soft tissue coverage, especially in settings with limited resources [6].

Advanced microsurgical techniques, such as free flaps and microvascular anastomosis, are considered the gold standard for hand reconstruction [7]. In settings like Yemen, where advanced microsurgical options such as free flaps are often inaccessible due to limited resources, the thin groin flap provides a feasible and reliable alternative. By focusing on the thin groin flap for hand reconstruction, this study aims to assess the surgical, functional, and aesthetic outcomes of a technique that can be performed quickly, with minimal donor-site morbidity, and at a lower cost compared to microsurgical procedures. These attributes make the thin groin flap especially significant for use in emergency situations, where time and resources are often limited. This research is crucial for expanding the knowledge base on reconstructive hand surgery in conflict zones, where the healthcare system is under significant strain. By evaluating the effectiveness of the thin groin flap in treating soft tissue defects of the hand, the study provided valuable insights that can guide future clinical practices and improve patient outcomes in resource-limited settings.

Furthermore, this study’s evaluation of the role of surgical magnification loops in improving precision and outcomes adds another dimension to understanding how simple yet effective tools can enhance the success of flap procedures in low-resource environments. The results of this study could lead to better surgical training, optimization of available resources, and, ultimately, improved quality of life for patients suffering from traumatic hand injuries in conflict zones. Therefore, this study aims to evaluate the surgical, functional, and aesthetic outcomes of the thin groin flap in hand reconstruction.

Hypothesis

The hypotheses of the study are as follows: There is no significant difference in functional outcomes between thin groin flaps and traditional groin flaps for hand reconstruction. A surgical approach using a magnification loupe provides superior functional and aesthetic outcomes compared to one without a loupe.

## Materials and methods

Study design and setting

This was a mixed retrospective-prospective study conducted in government and private hospitals in Sana'a, Yemen (Typical Police Hospital and Elite Hospital), which have reconstructive surgery units from January 2021 to December 2024. Those hospitals are considered the main reference hospitals for plastic surgery in Yemen.

Population and sample of the study

The study employed consecutive sampling to include all 25 patients presenting with traumatic or post-burn hand defects. By applying this total coverage approach, we ensured that every patient meeting the inclusion criteria received reconstructive flap surgery. Participants were selected based on a defined set of inclusion criteria, which required patients to be medically fit for surgical intervention, have a clinical indication for flap coverage, and provide formal consent to participate in the study.

To maintain the validity of the clinical outcomes, several exclusion criteria were applied. Patients were excluded if they presented with significant comorbidities that could independently influence surgical results, were lost to follow-up during the study period, or declined to participate. Given the specific nature of the condition, the final sample size included all 25 eligible patients who presented to the hospital and met the inclusion criteria during the designated study timeframe.

Surgical technique

Preoperative Preparation

Patient selection: Patients with hand and finger defects requiring soft tissue reconstruction are selected for this procedure. Key factors considered during selection include the size and location of the defect, vascular status of the patient, and the functional requirements for the hand post-reconstruction. A thorough clinical examination is conducted, accompanied by a Doppler ultrasound to assess the vascular supply of the groin flap. The surgical team discussed the expected outcomes, potential complications, and the staged nature of the procedure with the patient to ensure informed consent.

Preoperative marking: The thin groin flap is designed on the lower abdominal or groin region, ensuring that the flap is sufficiently thin and large enough for hand and finger reconstruction. The superficial circumflex iliac artery (SCIA), the primary vascular supply, is identified to ensure optimal circulation. The SCIA follows a constant oblique course in an ascending lateral direction, with a diameter of approximately 1.5-2 mm at its origin. Although the arterial and venous anatomy of the pedicle can present variations, its generally predictable structure makes it a reliable option for soft tissue reconstruction. Most surgeries are performed under general anesthesia. The contour of the flap is marked, and the axial vessel course is mapped, often with the help of Doppler ultrasound scanning.

Surgical Approach (Intraoperative Phase)

Stage 1: flap harvesting and initial transfer: A thin groin flap is raised, preserving the SCIA pedicle. Careful dissection is performed to maintain vascular integrity while ensuring the flap’s desired thinness for optimal hand coverage.

**Figure 1:**
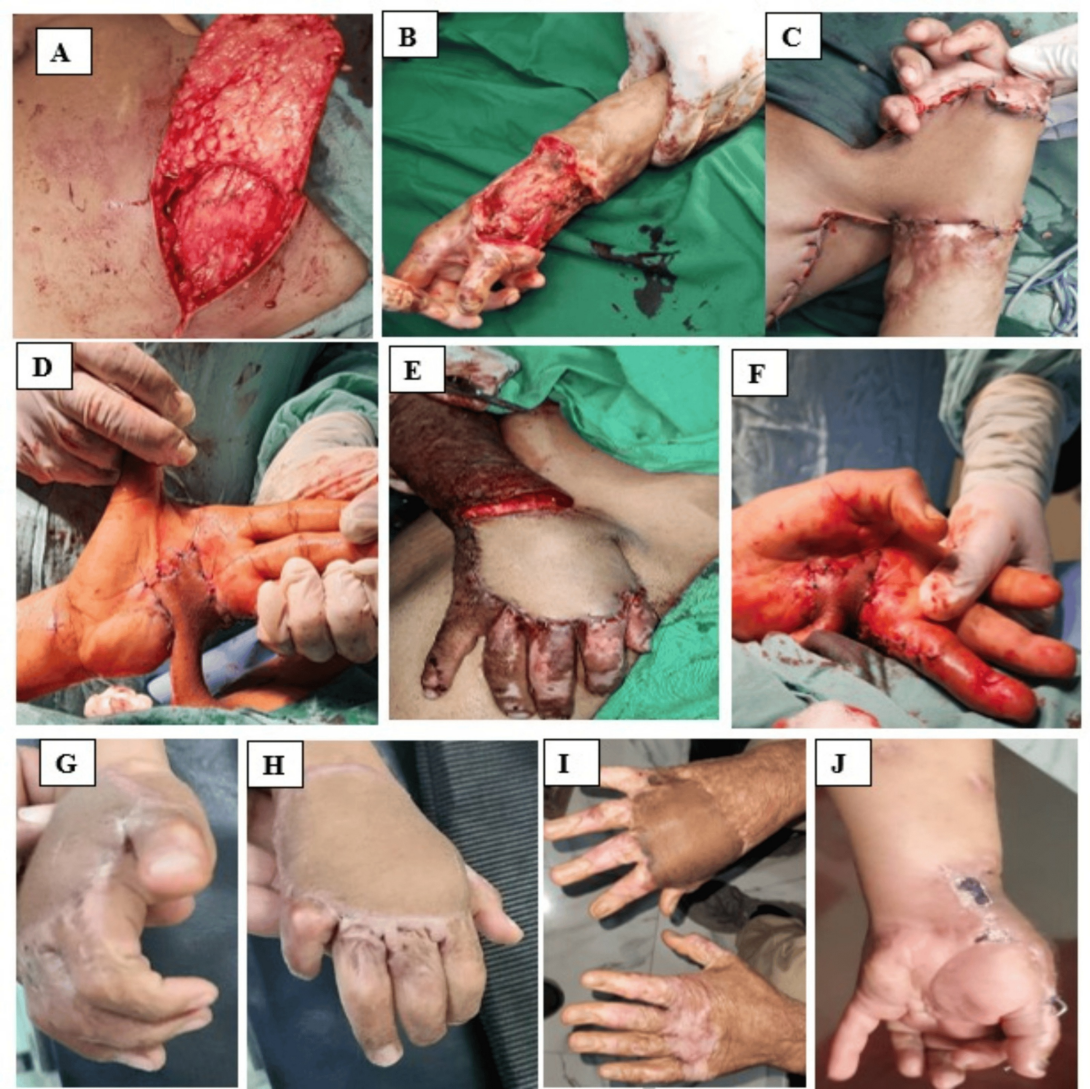
(A) A thin groin flap is raised; (B) wrist tissue defect prepared for flap coverage; (C-F) groin flap in the course of healing; (G-J) groin flap after detachment from pedicle.

The dissection begins at the anterior superior iliac spine region. After sequential transection of skin, subcutaneous tissue, and fascia, the flap is raised in a medial direction. If the patient is lean and the flap is thin, fascia may be spared in the initial dissection, beginning at the medial third of the groin, where the SCIA bends toward the femoral artery (Figure 1A). Additionally, in some cases, a surgical magnification loupe was used during the separation phase, while in others, it was not utilized.

Flap transfer: The harvested flap is transferred to the recipient site on the hand or fingers (Figure 1B) while remaining attached to the donor site in the abdomen. The flap is positioned and secured to the defect site, ensuring proper vascular integrity. The donor site is partially closed to allow for later division of the flap (Figures 1C-1F). The groin wound is generally sutured with slight tension. After surgery, the upper limb is secured with an adhesive bandage to prevent accidental repositioning and ensure the flap remains in place.

Monitoring and dressing: Flap perfusion is regularly monitored through capillary refill, temperature, and color assessments. Pain management and antibiotic prophylaxis are provided to prevent infection. The hand is immobilized using a splint to facilitate healing without trauma or tension. Patients are typically discharged 2-3 days after surgery once there are no signs of ischemia in the flap. In the early study period, low-molecular-weight heparin is administered for 10 days, followed by acetylsalicylic acid at a prophylactic dose for seven days in later periods. Wound healing is monitored at an outpatient clinic.

Stage 2: flap separation (after 21 days): pre-separation evaluation: Clinical assessment of the flap’s vascularization and integration is performed. Doppler ultrasound is used to confirm adequate blood supply from recipient vessels.

Flap separation surgery: The pedicle is carefully divided to establish independent circulation for the flap (Figures 1G-1J). The abdominal donor site is fully closed. In some cases, a surgical magnification loop is used for precise vascular dissection, while in others, standard techniques are applied.

Final inset and refinement: The flap is contoured and sutured to ensure optimal functional and aesthetic outcomes.

Study variables

The independent variables include age, sex, causes of injury, defect characteristics, and flap characteristics (design, dimensions, and intraoperative findings). The dependent variables include flap survival, complications, and functional outcomes.

Outcome measures

The primary outcome measures assessed in this study included flap survival, wound healing, functional outcomes, and patient satisfaction. Flap survival was determined by evaluating the presence of flap necrosis or loss. Wound healing was assessed by monitoring the time to complete epithelialization. Functional outcomes were evaluated using various scoring systems. Patient satisfaction was assessed through subjective measures, including patient-reported aesthetic outcomes and overall satisfaction with the procedure. The patients were assessed by the Disabilities of the Arm, Shoulder, and Hand (DASH) questionnaire after six months [8].

Data collection technique and tools

Data for this study were gathered through structured patient interviews, standardized clinical observation checklists, and longitudinal follow-up evaluations. The collected information initially focused on demographic profiles, including name, age, and sex, alongside a detailed medical history to identify comorbidities and chronic diseases. Clinical characteristics regarding the nature of the injury were meticulously documented, specifically focusing on defect parameters such as the underlying cause, size, anatomical location, and any associated injuries.

Furthermore, the study recorded comprehensive surgical details, including specific flap designs, harvested dimensions, and pertinent intraoperative findings. To evaluate the efficacy of the interventions, clinical outcomes were monitored by tracking complication rates and overall surgical success. Finally, patient-reported functional outcomes and levels of satisfaction were quantitatively assessed using the DASH questionnaire [8].

The DASH questionnaire is a 30-item clinical tool designed to evaluate upper extremity function across three domains: physical function (21 items), symptom severity (5 items), and social or role function (4 items). Patients rate their ability to perform daily activities, the intensity of symptoms like pain and weakness, and the impact of their condition on sleep and social interactions using a 5-point Likert scale ranging from 1 (no difficulty) to 5 (extreme difficulty). To calculate the final score, which ranges from 0 (no disability) to 100 (severe disability), the ratings are summed and transformed; however, a valid score requires at least 27 completed items, with missing values replaced by the mean of the existing responses.

Data processing and analysis

Data were entered into a statistical software package (SPSS; version 26; IBM Corp., Armonk, NY, USA). Descriptive statistics were used to summarize the demographic and clinical characteristics of the patients. Continuous variables were presented as means with standard deviations (SDs) or medians with interquartile ranges (IQRs), depending on the data distribution. Categorical variables were presented as frequencies and percentages. The primary outcomes, including flap survival, wound healing, functional outcomes, and patient satisfaction, were analyzed and reported by using the appropriate statistical methods (Fisher's exact test, Kruskal-Wallis test, and Spearman correlation). Results are presented in tables and charts for clarity.

Ethical considerations

Ethical approval was obtained from the Yemeni Board of Plastic Surgery in Yemen. Besides, administrative approval was obtained from the affiliated hospitals, Sana’a, Yemen. Written informed consent was received from all participants, who were informed that their participation was voluntary and that their information was used for research purposes and was kept strictly confidential.

## Results

Table 1 shows that this study involved 25 patients undergoing thin groin flap reconstruction for hand and finger defects. The median age was 27.0 years (IQR 22.0-40.0), with a slight male predominance (56.0%, n = 14). The median defect size was 7.0 cm² (IQR 6.0-7.0), and the median time from initial injury to surgical reconstruction was 7.0 days (IQR 5.0-16.0). The vast majority of procedures (88.0%, n = 22) were performed using surgical magnification loupes. The median duration of surgery was 90.0 minutes (IQR 90.0-120.0), with a median postoperative hospital stay of 5.0 days (IQR 3.0-5.0). The primary causes of injury were evenly split between non-gunshot trauma (40.0%, n = 10) and burns (40.0%, n = 10), with high-energy gunshot injuries accounting for the remainder (4, 16.0%).

**Table 1:** Demographic and clinical characteristics (n = 25). IQR: interquartile range; cm²: centimeters squared

Characteristic	Frequency (n)/value	Percentage (%)
Age (years), median (IQR)	27.0 (22.0-40.0)	-
Sex		
Male	14	56.0%
Female	11	44.0%
Cause of injury		
Trauma (non-gunshot)	10	40.0%
Burn	10	40.0%
Gunshot	4	16.0%
Other (tumor excision)	1	4.0%
Defect size (cm²), median (IQR)	7.0 (6.0-7.0)	-
Time from injury to surgery (days), median (IQR)	7.0 (5.0-16.0)	-
Surgical loupe use		
Used	22	88.0%
Not used	3	12.0%
Surgery duration (minutes), median (IQR)	90.0 (90.0-120.0)	-
Hospital stay (days), median (IQR)	5.0 (3.0-5.0)	-

Table 2 reveals that partial flap necrosis occurred in one patient (4.0%), resulting in a complete flap survival rate of 96.0%. An overall success rate, defined as the absence of any complication at either the recipient or donor site, was achieved in 80.0% of cases (n = 20). Complications at the recipient site included infection (8.0%, n = 2), partial necrosis (4.0%, n = 1), and swelling (4.0%, n = 1). Donor-site complications were also recorded, with four different issues each occurring in one patient (4.0%). Functional outcomes, assessed in 24 patients, were generally favorable. The median DASH score was 10.0 (IQR 5.0-25.0). More than half of the patients (52.0%, n = 13) were satisfied with the final aesthetic outcome, while nine (36.0%) were neutral and three (12.0%) were dissatisfied.

**Table 2:** Postoperative complications and outcomes. IQR: interquartile range; DASH: Disabilities of the Arm, Shoulder, and Hand

Outcome measure	Frequency (n)	Percentage (%)
Recipient-site complications		
Infection	2	8.0%
Partial flap necrosis	1	4.0%
Swelling	1	4.0%
Donor-site complications		
Wound dehiscence	1	4.0%
Hypertrophic scarring	1	4.0%
Infection	1	4.0%
Swelling or redness	1	4.0%
Overall success rate (no complications)	20	80.0%
DASH score (0-100), median (IQR)	10.0 (5.0-25.0)	-
Aesthetic satisfaction		
Satisfied	13	52.0%
Neutral	9	36.0%
Dissatisfied	3	12.0%

Table 3 shows that the use of surgical magnification loupes was associated with markedly superior outcomes. The complication rate in the loupe group was 9.1% (n = 2), whereas all patients in the no-loupe group experienced at least one complication (100.0%, n = 3). This difference was statistically significant (Fisher's exact test, χ² = 25.00, p = 0.028). Functionally, the loupe group demonstrated significantly better results, with a median DASH score of 10.0 (IQR 5.0-25.0) compared to 50.0 (IQR 50.0-50.0) in the no-loupe group (Mann-Whitney U = 0.0, p = 0.018). Furthermore, the use of loupes was associated with a significantly shorter postoperative hospital stay (median 4.5 days vs. 15.0 days; Mann-Whitney U = 0.0, p = 0.004). No significant difference was found in the duration of surgery between the two groups (p = 0.716).

**Table 3:** Comparison of outcomes based on the use of surgical magnification loupes. IQR: interquartile range; DASH: Disabilities of the Arm, Shoulder, and Hand *Statistically significant

Outcome	Loupe group (n = 22)	No-loupe group (n = 3)	Test statistic	p-value
Any complication, n (%)	2 (9.1%)	3 (100.0%)	χ^2^ = 25.00	0.028*
DASH score, median (IQR)	10.0 (5.0-25.0)	50.0 (50.0-50.0)	U = 0.00	0.018*
Hospital stay (days), median (IQR)	4.5 (3.0-5.0)	15.0 (14.0-16.0)	U = 0.00	0.004*
Surgery duration (min), median (IQR)	90.0 (90.0-120.0)	120.0 (105.0-120.0)	U = 28.50	0.716

**Figure 2:**
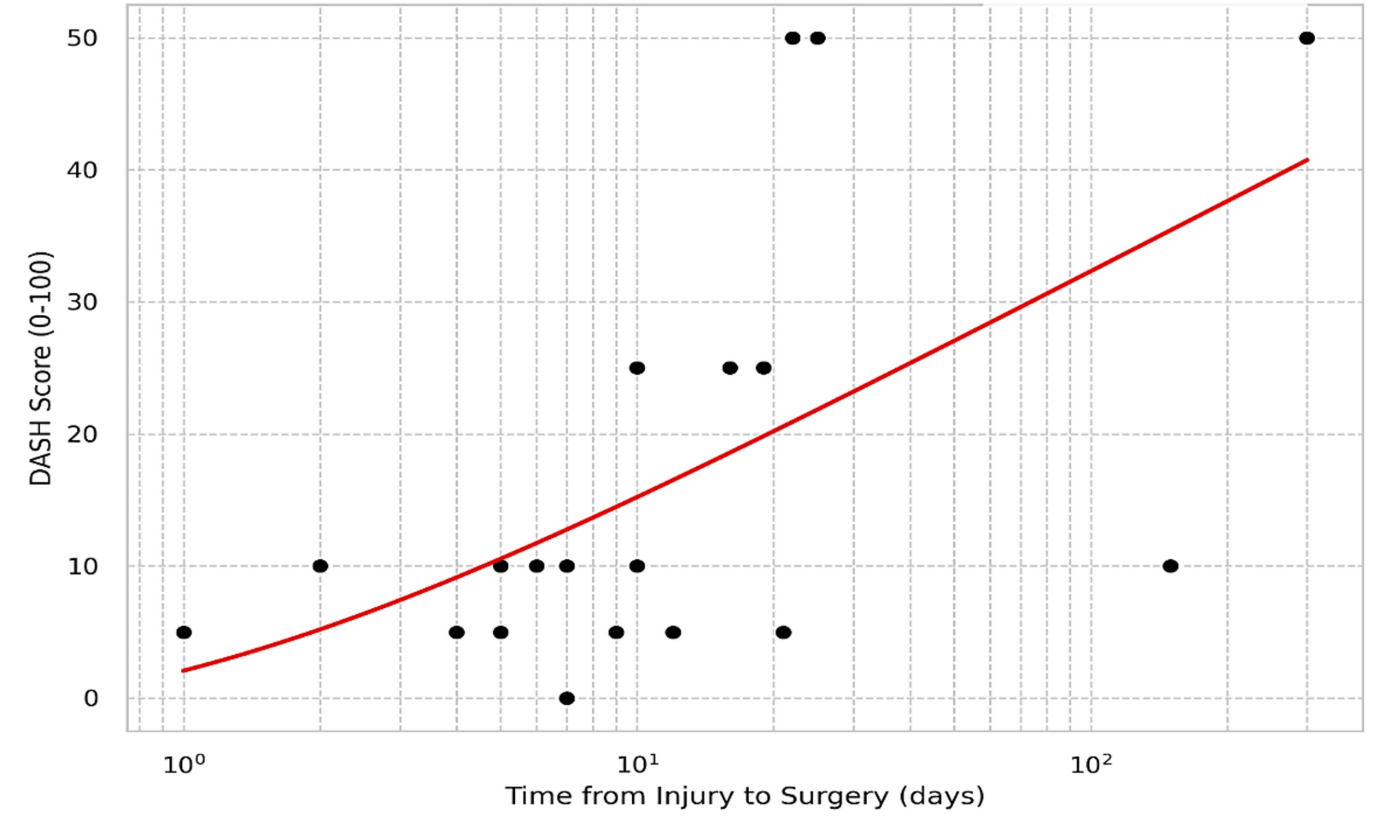
Relationship between the time from injury to surgery and the postoperative DASH score (n = 24). DASH: Disabilities of the Arm, Shoulder, and Hand

Table 4 reveals that the cause of injury significantly influenced outcomes. Patients with gunshot injuries had significantly worse functional outcomes compared to those with burn or trauma injuries (Kruskal-Wallis test, p = 0.005). The presence of associated injuries (tendon, nerve, or bone fracture) at the time of presentation was another critical determinant of outcome. Patients with associated injuries (n = 12) had significantly higher DASH scores, indicating greater disability, compared to patients with isolated soft tissue defects (n = 13) (median DASH 10.0 vs. 5.0; Mann-Whitney U = 31.0, p = 0.013). Furthermore, correlation analysis revealed that clinical timing and injury severity were strongly linked to function. As shown in Figure 2, a significant positive correlation was observed between the delay from injury to surgery and higher postoperative DASH scores. A longer delay between the initial injury and surgical reconstruction was strongly associated with poorer functional outcomes (r’s = 0.518, p = 0.009). A larger initial defect size was also significantly correlated with worse DASH scores (r’s = 0.422, p = 0.040). The duration of the surgical procedure itself, however, showed no significant correlation with the final functional outcome (p = 0.206).

**Table 4:** Analysis of predictors for functional outcome (DASH score). DASH: Disabilities of the Arm, Shoulder, and Hand; IQR: interquartile range; r: correlation coefficient; cm²: centimeters squared *Statistically significant (p < 0.05)

Factor	Group/variable	Median DASH (IQR) or correlation (r)	Test statistic	p-value
Cause of injury	Burn (n = 10)	5.0 (5.0-10.0)	H = 10.45	0.005*
	Trauma (n = 10)	10.0 (5.0-10.0)		
	Gunshot (n = 4)	37.5 (25.0-50.0)		
Associated injuries	None (n = 13)	5.0 (5.0-10.0)	U = 31.00	0.013*
	Present (n = 12)	10.0 (10.0-25.0)		
Time (injury to surgery)	Continuous (days)	r = 0.518	r = 0.518	0.009*
Defect size (cm^2^)	Continuous (cm^2^)	r = 0.422	r = 0.422	0.040*
Duration of surgery	Continuous (min)	r = 0.268	r = 0.268	0.206

## Discussion

The present study included 25 patients who underwent thin groin flap reconstruction for hand defects. The study had a median age of 27.0 years (IQR 22.0-40.0), with a slight male predominance (56.0%, n = 14). The primary causes of injury were evenly split between non-gunshot trauma (10, 40.0%) and burns (10, 40.0%), with high-energy gunshot injuries accounting for the remainder (4, 16.0%). The median defect size was 7.0 cm² (IQR 6.0-7.0), and the median time from initial injury to surgical reconstruction was 7.0 days (IQR 5.0-16.0).

The present study reported a high complete flap survival rate of 96.0%, with partial flap necrosis occurring in only one patient (4.0%). The overall success rate, defined as the absence of any complication at either the recipient or donor site, was 80.0%. These findings are consistent with the literature, which generally reports favorable outcomes for groin flaps in hand reconstruction. For example, a study by Abualhaj et al. reported that flap success was achieved in 91.3% among hand degloving injury cases [9]. Naalla et al. reported that flap healing was achieved in a total of 99% of cases, with total flap necrosis occurring in only one case [10]. Jabaiti et al. reported that flap healing was observed in 29 patients (85%) [11]. Also, Żyluk reported no postoperative complications in 74% of patients, with primary healing of wounds and complete healing of flaps [12]. In addition, Altuntas et al. noted that all flaps survived [13].

The current study reported complications such as recipient-site complications, including infection (8.0%) and swelling (4.0%), while various donor-site complications occurred in 4.0% of patients. These results align with previous studies. For instance, Naalla et al. reported partial flap necrosis in 15 patients (7%), surgical site infection in 11 patients (5%), flap dehiscence in five patients (2.4%), and total flap necrosis in four patients (2%) [10]. Abualhaj et al. noted postoperative complications in 34.8% of patients, including partial flap necrosis (26.1%), wound dehiscence (13.0%), and infection (4.3%) [9]. Also, Żyluk reported that 26% of their patients experienced complications, including whole flap necrosis (one patient), marginal flap necrosis (three patients), partial flap detachment (two patients), and groin wound infection (two patients) [12]. This suggests a slightly higher complication rate in Żyluk's study compared to the present one, although direct comparison is challenging due to differences in complication definitions and patient cohorts. Altuntas et al. noted complications such as wound dehiscence and infection in four patients in their pedicled flap group, while no flap-related complications were seen in their free flap group, highlighting the potential influence of the flap type on complication rates [13]. The findings by Altuntas et al. align with our results because our study has one patient complaining of partial necrosis, which was managed, and the flap was successful. The low rate of total flap necrosis in our study aligns with the general understanding of the groin flap as a reliable and safe option for hand reconstruction.

Functional outcomes in the present study, assessed using the DASH score, were generally favorable, with a median score of 10.0 (IQR 5.0-25.0), indicating low levels of disability. Patient satisfaction with the final aesthetic outcome was also positive, with 88.0% of patients reporting neutral (36%) and satisfied (52%) responses, and 12.0% reporting dissatisfied. These results can be compared with other studies that utilized the DASH questionnaire. Abualhaj et al. reported that most of the patients (91.3%) had good functional scores, with a mean Quick DASH score of 18.1 (SD = 14.1) [9]. Also, Altuntas et al. reported significantly higher DASH scores in their pedicled flap group (92 in subacute and 72 in chronic stages) compared to their free flap group (52 and 24, respectively), indicating greater disability with pedicled flaps. A direct comparison of absolute DASH scores is difficult due to varying patient populations and follow-up periods. Furthermore, Żyluk reported an average Quick DASH score of 21.5 points, corresponding to a moderate loss in dexterity, with some patients experiencing significantly impaired hand function (Quick DASH score above 40 points). Our median DASH score of 10.0 is considerably lower than Żyluk's Quick DASH average, suggesting a better overall functional outcome in our cohort. This difference might be attributed to various factors, including patient selection, surgical techniques, and rehabilitation protocols.

The aesthetic satisfaction reported in our study (88.0% satisfied or neutral) is a crucial aspect of reconstructive surgery. This result is similar to a previous study in Jordan that revealed that the aesthetic outcomes were rated as good or neutral in over 90% of the cases [9]. While most previous studies did not explicitly detail aesthetic satisfaction rates, emphasis on patient-reported outcomes in Altuntas et al. [13] and the discussion of patient comfort in Żyluk [12] underscore the importance of holistic outcomes beyond mere flap survival. The present study's findings contribute valuable data on patient perception of aesthetic results, which is often a key determinant of overall satisfaction in hand reconstruction.

A notable finding in the present study was the significant positive impact of surgical magnification loupes on outcomes. The complication rate in the loupe group was substantially lower (9.1%) compared to the no-loupe group (100.0%), a statistically significant difference (Fisher's exact test, p = 0.028). Furthermore, the loupe group demonstrated significantly better functional outcomes (median DASH score of 10.0 vs. 50.0 in the no-loupe group; Mann-Whitney U = 0.0, p = 0.018) and a shorter postoperative hospital stay (median 4.5 days vs. 15.0 days; Mann-Whitney U = 0.0, p = 0.004). This highlights the critical role of magnification in improving surgical precision and, consequently, patient outcomes. While the provided previous studies [7,12-14] discuss various aspects of groin flap reconstruction, none explicitly detail the impact of surgical magnification loupes on outcomes. This makes the present study's findings particularly valuable, as they provide direct evidence supporting the use of magnification in this specific context. The improved complication rates, functional recovery, and reduced hospital stay observed in the loupe group strongly suggest that enhanced visualization contributes to more precise dissection, better vascular anastomosis (if applicable), and overall superior surgical execution. This finding could inform best practices in hand reconstructive surgery, advocating for the routine use of magnification to optimize patient outcomes.

The present study identified that postoperative DASH scores are primarily influenced by the nature of the injury and timing of treatment. Gunshot wounds, associated injuries (nerve, tendon, or bone), larger defect sizes, and delayed surgical reconstruction all significantly correlate with higher disability. These findings resonate with general principles in reconstructive surgery, where injury severity and timing of intervention are known to influence outcomes. While the previous studies did not specifically analyze these exact predictors in the same manner, they provide indirect support for these observations. For instance, Al-Qattan and Al-Qattan acknowledge that complex defects and severe injuries (such as high-voltage electric burns or mutilating hand injuries) may necessitate specific reconstructive approaches, which could inherently be associated with different functional outcomes [15]. Also, Żyluk noted that the weakest functional results were obtained in patients where flap coverage involved digits and simultaneous tendon reconstruction was required, which aligns with our finding that associated injuries negatively impact functional outcomes. The groin flap is a very versatile flap for coverage because of its supple skin and robust blood supply. However, the cost of donor-site morbidity should be minimized through proper planning and meticulous surgical technique [5].

The strong correlation between surgical delay and poorer functional outcomes in our study underscores the importance of timely intervention in hand reconstruction. This is a critical point for clinical practice, suggesting that efforts to reduce time from injury to surgery could lead to improved patient recovery. Similarly, the impact of defect size on functional outcome highlights the challenges associated with reconstructing larger defects, which may require more complex procedures and potentially lead to less optimal functional restoration.

Strengths and limitations

This study possesses several notable strengths that contribute to the existing literature on hand reconstruction. By utilizing both prospective and retrospective data over a four-year period, the study provides a comprehensive look at the thin groin flap's performance in a real-world clinical setting. A significant strength is the subgroup analysis regarding magnification loupes, which offers valuable evidence on how microsurgical aids can directly reduce complication rates and improve functional recovery. Furthermore, the use of a validated instrument like the DASH questionnaire ensures that the functional outcomes were measured using a standardized, internationally recognized metric, allowing for better comparison with other global studies.

Despite these strengths, the study has certain limitations that should be considered. The sample size of 25 patients is relatively small, which may limit the generalizability of the findings and the statistical power to detect minor differences in outcomes. Furthermore, the study lacked a comparative control group, as it was designed as a consecutive case series to evaluate the specific technical reliability of the groin flap rather than comparing it to alternative reconstructive techniques. Additionally, the study was conducted across only two centers in a specific geographic region (Sana'a, Yemen), which may reflect localized surgical practices or patient demographics that differ elsewhere. Finally, the study primarily focused on short-to-medium-term outcomes; a longer follow-up period would be beneficial to assess the long-term aesthetic refinement and late-stage functional improvements of the thin groin flap.

## Conclusions

This study evaluated the outcomes of thin groin flap reconstruction in 25 patients with hand and finger defects. Our findings demonstrate high flap survival rates and generally favorable functional outcomes. Furthermore, we identified several critical predictors of functional outcome, including the cause of injury, the presence of associated injuries, the time from injury to surgery, and the initial defect size. These insights underscore the importance of early intervention and meticulous surgical planning, particularly in cases of severe trauma. While the groin flap remains a versatile and reliable option, the judicious use of surgical magnification and a comprehensive understanding of prognostic factors can further optimize patient outcomes in hand reconstruction. A larger multicenter prospective study with long-term follow-up is recommended to validate the durability of functional and aesthetic outcomes and to detect late complications.

## References

[1] Arroyo-Berezowsky C, Quinzaños-Fresnedo J (2021). Epidemiology of hand and wrist injuries treated in a reference specialty center over a year. Acta Ortop Mex.

[2] Orozco-Grad1os JJ, Cordova JC, Garcia Garcia JA, Baez Armenta DY, Gonzalez AA, Galvis DC (2023). Groin flap for reconstruction of traumatic degloving hand injury: a report of 5 cases. World J Plast Surg.

[3] Lai YM, Eong JT, Tan BK (2024). Pedicled SCIA and SIEA mega groin flap-a staged reconstructive approach for large forearm defects. J Plast Reconstr Surg.

[4] Iskander S, Halbesma G, Hoogbergen MM, Young-Afat D, Veldhuizen IJ (2025). Comprehensive assessment of specific patient-reported outcome measures for hand and wrist conditions in adults: a scoping review. JPRAS Open.

[5] Adeel I, Ali G, Mirza TI, Malik MJ (2021). Groin flap; a simple and versatile option for coverage of hand defects. Med Forum.

[6] Hassan SM (2024). Outcome of paraumbilical perforator flap for coverage of soft tissue deficiency around the forearm and hand. The Insight.

[7] Ziegler B, Hundeshagen G, Will PA, Bickert B, Kneser U, Hirche C (2020). Role, management, and outcome of free flap reconstruction for acute full-thickness burns in hands. Ann Plast Surg.

[8] Hudak PL, Amadio PC, Bombardier C (1996). Development of an upper extremity outcome measure: the DASH (disabilities of the arm, shoulder and hand) [corrected]. The Upper Extremity Collaborative Group (UECG). Am J Ind Med.

[9] Abualhaj S, Al-Zamer YS, Al-Shadfan L, Abualhaj M, Aloun A, Massad BJ (2025). Evaluating pedicled groin flap reconstruction in hand degloving injuries: a retrospective analysis. J Hand Surg Glob Online.

[10] Naalla R, Chauhan S, Dave A, Singhal M (2018). Reconstruction of post-traumatic upper extremity soft tissue defects with pedicled flaps: an algorithmic approach to clinical decision making. Chin J Traumatol.

[11] Jabaiti S, Ahmad M, AlRyalat SA (2020). Reconstruction of upper extremity defects by random pedicle abdominal flaps: is it still a valid option?. Plast Reconstr Surg Glob Open.

[12] Żyluk A (2022). Outcomes of coverage of the soft tissue defects in the hand with a groin flap. Pol Przegl Chir.

[13] Altuntas SH, Dilek OF, Gurdal O, Uslusoy F, Aydin MA (2023). The use of groin flap for hand defects: which should be prior, free or pedicled, based on patient-reported outcomes?. Selcuk Med J.

[14] Jokuszies A, Niederbichler AD, Hirsch N, Kahlmann D, Herold C, Vogt PM (2010). The pedicled groin flap for defect closure of the hand (Article in German). Oper Orthop Traumatol.

[15] Al-Qattan MM, Al-Qattan AM (2016). Defining the indications of pedicled groin and abdominal flaps in hand reconstruction in the current microsurgery era. J Hand Surg Am.

